# High optical enhancement in Au/Ag alloys and porous Au using Surface-Enhanced Raman spectroscopy technique

**DOI:** 10.1038/s41598-021-84093-0

**Published:** 2021-02-25

**Authors:** C. Awada, C. Dab, M. G. Grimaldi, A. Alshoaibi, F. Ruffino

**Affiliations:** 1 Department of Physics, College of Science, King Faisal University, P.O. Box: 400, Al-Ahsa, 31982 Saudi Arabia; 2 Département de Chimie, Université de Montréal, Campus MIL, Montréal, QC H2V 0B3 Canada; 3 Dipartimento di Fisica e Astronomia “Ettore Majorana”-Università di Catania and MATIS CNR-IMM, via S. Sofia 64, 95123 Catania, Italy

**Keywords:** Nanoscale devices, Nanoscale materials, Synthesis and processing

## Abstract

We report high optical enhancement in Ag/Au alloys and porous gold nanostructures using Surface Enhanced Raman Spectroscopy (SERS) technique. Scanning electron microscopy investigation shows the formation of Ag/Au alloys particles during irradiation of Ag–Au bilayer deposited on FTO (SnO_2_:F) substrate by laser fluency equal to 0.5 J/cm^2^ or 1.0 J/cm^2^ with 12 ns laser pulse duration. The dealloying process of these Au–Ag alloy particles leads to the formation of Au nanoporous particles. The obtained nanostructures were studied with SERS and revealed a promising enhancement factor in porous Au nanostructure and tunability of localized surface plasmon resonance. The highly dense strong hot spots and large specific area in porous structure of gold nanostructures is the origin of the highly enhancement factor observed experimentally and theoretically. A very good agreement between simulation and experimental results was found confirming the potential of Au/Ag alloys and particularly porous gold nanostructure in SERS application.

## Introduction

Material nano processing to fabricate micro and nano metallic structures (nanoparticles, nanoholes, nanofibers) presents a challenging area especially in the material science domain to fulfill the needed dimensions, structure, optical properties. Recently, several methodologies have been carried out in order to control the structural properties of metallic nanostructure, their densities and atomic interaction such as ultrafast lasers that opens a new ways for material treatment^1,2^. From nanosecond to femtosecond pulsed laser, many materials have been investigated in nanophononics, nanoelectronics and integrated optics^3–6^. Among these materials, silver and gold nanostructures assembled onto a transparent conducting oxide substrate like indium tin oxide have been widely used in surface enhanced Raman spectroscopy (SERS) application thanks to their localized surface plasmon resonance (LSPR) properties^7–9^. Besides, fluorine doped tin oxide FTO (SnO_2_:F) substrate presents interesting features. FTO film is a wide bandgap semiconductor (3.80 eV) with high transmittance in the visible and near infrared (NIR) regions of the electromagnetic spectrum. FTO is also a highly degenerate semiconductor with low electrical resistivity (6.71 × 10^−3^ Ω cm)^10^ typically used as electrodes for solar cells and photovoltaic applications^11–15^. Silver and gold nanoparticles are characterized by their strong enhancement factor and their performance relies on the excitation of localized surface plasmon resonance (LSPR) that alter, confine and reinforce light-matter^16,17^. As a result, researches focus on preparing ideal noble metallic nanostructures as SERS substrates for tuning LSPR resonance. LSPR provide a high density of electrons and low losses from the visible to NIR regions and are highly sensitive to the shape anisotropy, size and surface chemistry of the metallic nanoparticles. The resonance wavelength of LSPR may be tuned depending on the size of nanoparticles^18–20^, optical and physical properties (the refractive index, permittivity) of the corresponding medium. The literature works investigated the SERS effect in nanoporous Au films or leafs^21–24^ and in Au/Ag alloy nanostructures^25,26^ and, more recently, large interest is captured by the study of plasmonic and SERS in porous Au nanodisks and particles^27–30^ effects . In particualr, in this work we highlight that the mixing of silver and gold nanoparticles on fluoride tin oxide substrate could be successfully used in plasmonic solar cells and SERS leading to a potential enhancement factor and selective photon absorption^31–33^, and through a combination of both experiments and simulations, we investigate SERS enhancement tunability in Ag/Au alloys and porous Au nanostructures and LSPR tunability in porous Au nanostructures.

Based on such considerations, we showed in this study the formation and evolution of Au/Ag alloys and porous nanostructure by nanosecond laser irradiation of Au(7.7 nm) and Ag(50 nm) bilayer deposited on fluorine doped tin oxide. Scanning electron microscopy (SEM) measurements indicates different top-view scans obtained from the FTO surface after being covered by the Au and Ag films to the fabrication of Au/Ag alloys after laser irradiation (0.5 J/cm^2^ and 1.0 J/cm^2^) and porous Au after dealloying in HNO_3_. Then, we performed surface enhanced Raman intensity measurement on the different sample for a comparative study between the different structures. The porous gold nanostructures show a strong enhancement factor compared to their Au/Ag alloys counterparts confirming their potential application in SERS. Finally, we confirmed the obtained enhancement factor on Au/Ag alloys and porous Au using finite element method based COMSOL Multiphysics and we found a very good agreement between experiment and simulation in term of enhancement factor quantification.

## Results and discussions

Supplementary Fig. S1a in the supporting information, reports the plan-view SEM image of the bare FTO substrate (before Au and Ag depositions). The FTO surface presents a rough surface composed by extended touching pyramids corresponding to the FTO columnar grains formed during the FTO deposition on quartz^34^.

Similarly, Supplementary Fig. S1b in the supporting information reports the plan-view SEM image of the FTO surface after being covered by the Au and Ag films. The Au/Ag bilayer conformally covers the substrate surface. The bilayer surface shows the typical nanogranular rough morphology of metal films in the late stage of growth, after metal clusters nucleation and growth, coalescence and percolation, and, finally, voids filling^35,36^.

**Figure 1:**
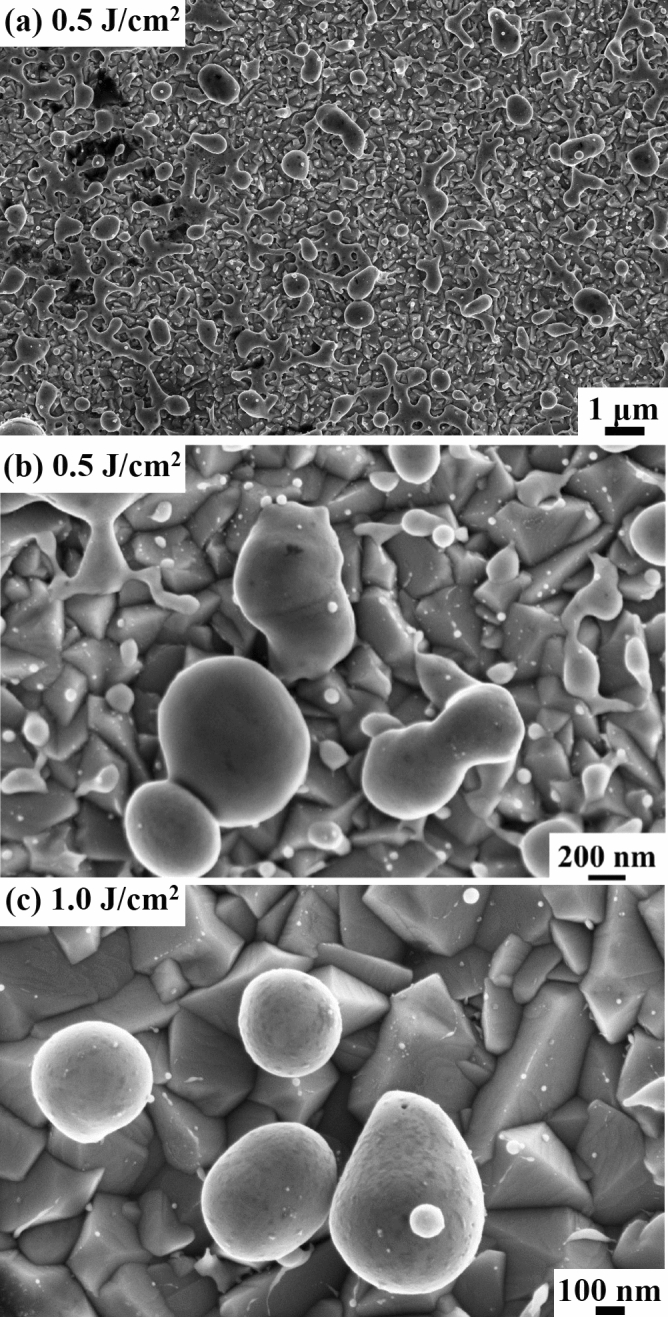
Representative SEM images of the alloy Ag–Au particles obtained on the FTO surface after irradiating the Ag–Au bilayer by laser fluence: (**a**,**b**) 0.5 J/cm^2^, with increasing magnification from (**a**) to (**b**), (**c**) 1.0 J/cm^2^.

Figure 1a,b show representative SEM image of the alloy Ag–Au particles obtained on the FTO surface after irradiating the Ag–Au bilayer by laser fluence 0.5 J/cm^2^, with increasing magnification from (a) to (b). We can observe that the dewetted particles are homogeneously distributed over the entire FTO surface. Large and small particles are present as arising form the stochastic nature of the dewetting process. The situation is similar for the sample obtained by the 1.0 J/cm^2^ laser irradiation: as an example, Fig. 3c shows a representative SEM image (high-magnification) of the alloy Ag–Au particles obtained in this case.

**Figure 2:**
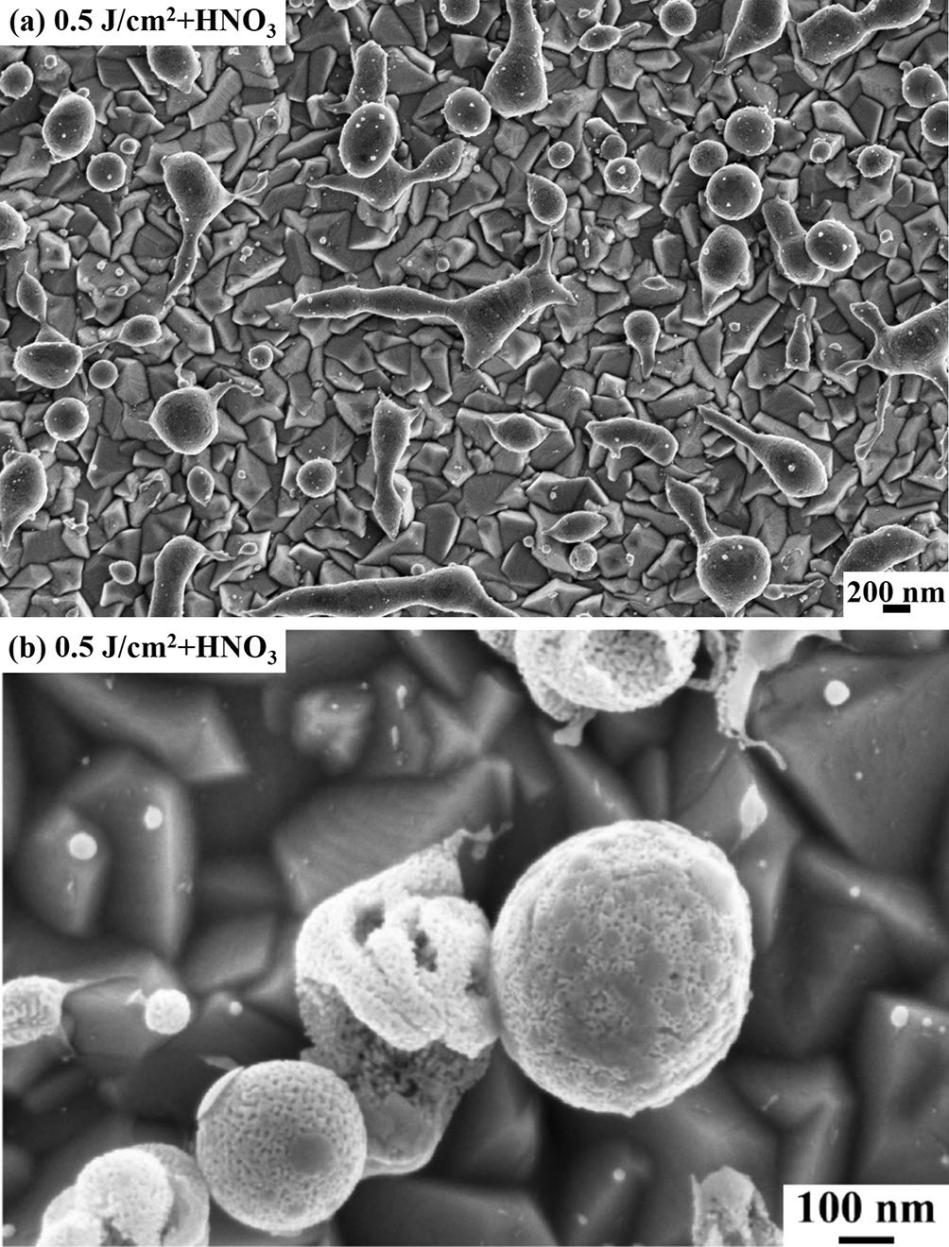
Representative SEM images [with increasing magnification from (**a**) to (**b**)] of the nanoporous Au particles obtained by HNO_3_ dealloying of the alloy Au–Ag particles obtained on the FTO surface after irradiating the Ag-Au bilayer by laser fluence 0.5 J/cm^2^.

Figure 2 reports a representative SEM image of the nanoporous Au particles obtained by the dealloying process of the Ag–Au alloy particles obtained by laser irradiation of the Ag–Au bilayer at 0.5 J/cm^2^, with increasing magnification from (a) to (b). On a large scale, wecan observe that the HNO_3_ etch does not impact on the particles distribution over the FTO surface. It only causes the etching of the Ag atoms from the Ag–Au alloy particles determing the formation of the Au nanoporous particles.

**Figure 3:**
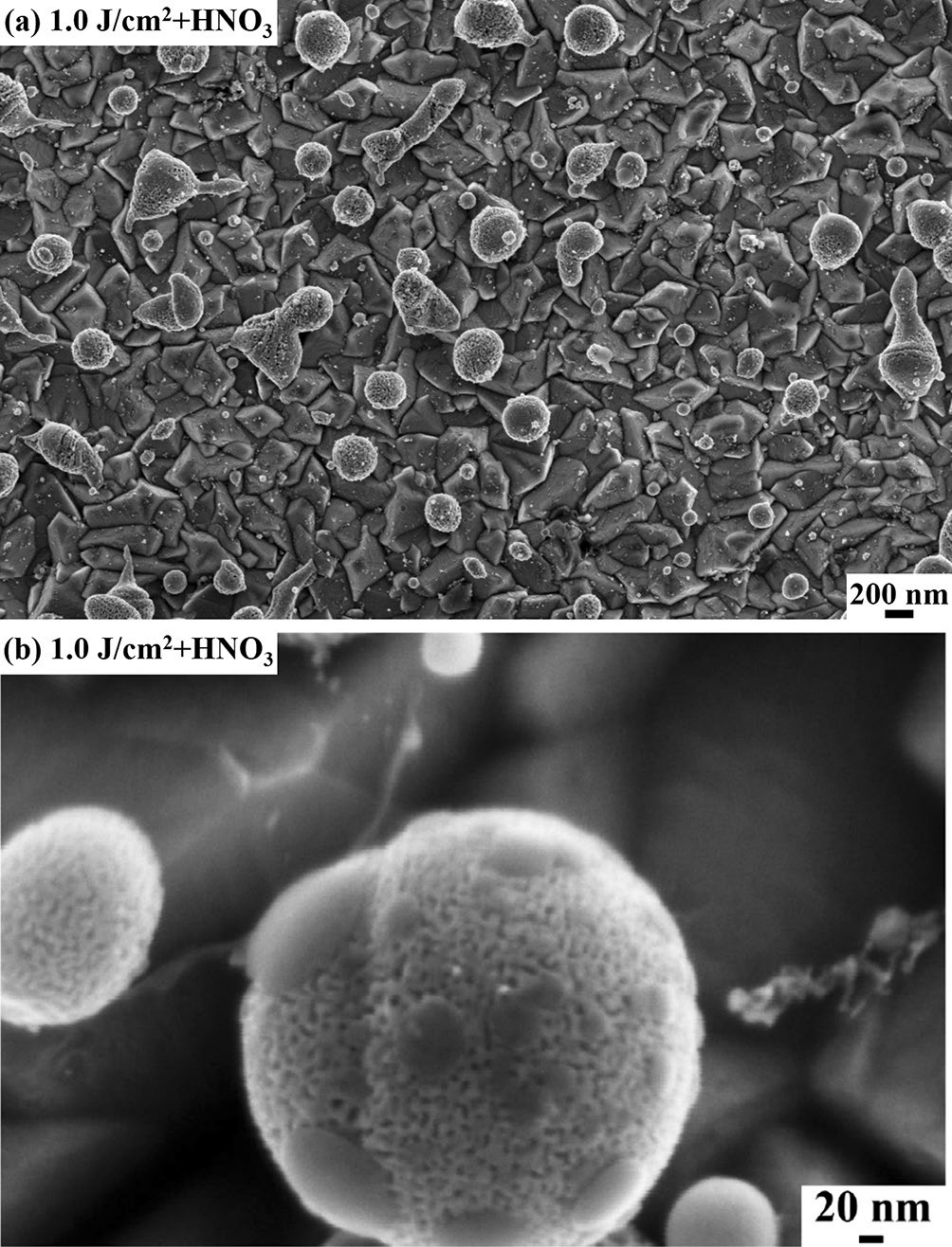
Representative SEM images [with increasing magnification from (**a**) to (**b**)] of the nanoporous Au particles obtained by HNO_3_ dealloying of the alloy Au–Ag particles obtained on the FTO surface after irradiating the Ag-Au bilayer by laser fluence 1.0 J/cm^2^.

Figure 3 reports a representative SEM image of the nanoporous Au particles obtained by the dealloying process of the Ag–Au alloy particles obtained by laser irradiation of the Ag-Au bilayer at 1.0 J/cm^2^, with increasing magnification form (a) to (b).

From a general point of view, the Au/Ag bilayer deposited on the FTO substrate, after the nanosecond laser irradiation melts and reacts to form the alloy. Regarding this last point, the mixing of the two metals occurs within the molten time and the mixing is promoted by the very high inter-diffusion coefficient of the metals in the liquid state^37^ and the complete miscibility of Au and Ag over all range of concentration^38^. Concerning the dewetting process, it starts immediately upon melting^8,39,40^.

Concerning molten metal films on non-wetting substrates, the main structuring mechanism is the so-called spinodal dewetting which implies the enhancement of film thickness fluctuations. A real deposited metal film presents natural height fluctuations, giving rise to its surface roughness and during thermal processes these fluctuations evolve by increasing their amplitude. However, this effect is contrasted by the restoring surface tension.

The overall film evolution is determined by the dominant effect. In particular, the molten metal film becomes unstable in the condition for which the van der Waals forces between the film atoms and the substrate atoms are smaller than the cohesive forces between the atoms forming the film. The film is, then, in a condition of thermodynamic instability and it breaks in droplets (ideally of spherical shape) to minimize the totale surface and interface energy of the system. The Ag atoms are removed from the Ag–Au particles by a simple etching process (dealloying) in 70% HNO_3_, being Ag easily oxidized to a nitrate salt by HNO_3_ whereas Au is not. The Ag chemical selective dissolution leads to the self-assembly of Au atoms at alloy/electrolyte interface resulting in final porous structure for the particles, and the process is controlled by the Au atoms diffusion at the alloy/electrolyte interface^24^.

**Figure 4:**
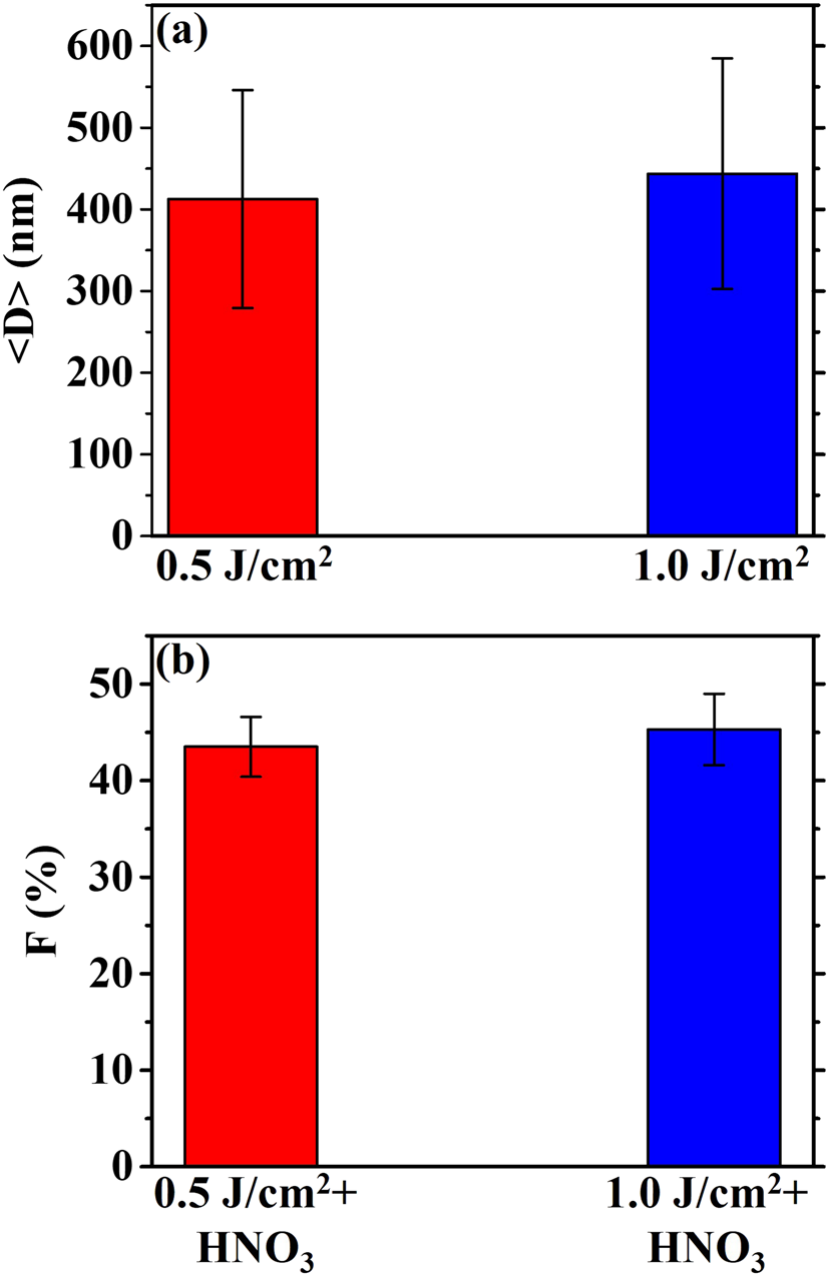
(**a**) Plot summarizing the measured average diameter < D > for the AuAg alloy particles obtained by the laser irradiation at 0.5 J/cm^2^ and 1.0 J/cm^2^. (**b**) Plot summarizing the measured porosity fraction F (expressed as %) for the nanoporous Au particles obtained by the laser irradiation at 0.5 J/cm^2^ and 1.0 J/cm^2^ of the AuAg bilayer and followed by the Ag removal by HNO_3_ etching.

Figure 4 summarizes in (**a**) the average diameter < D > of the nanoporous Au particles and in (**b**) the average porosity fraction F for the two samples. For both samples, the avergage diameter of the particles is around 400 nm and the average porosity fraction is around 44%.

**Figure 5:**
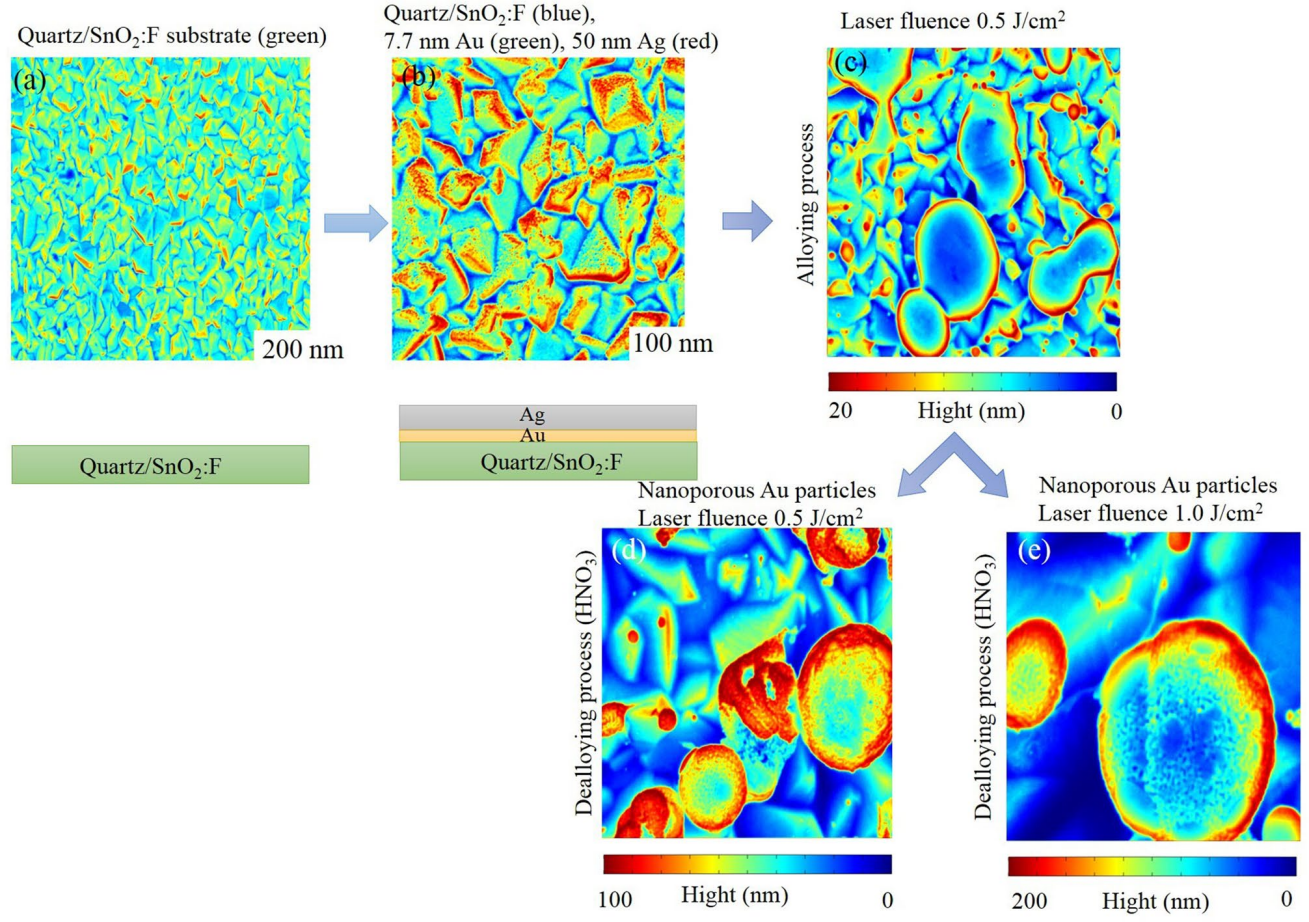
Schematic (top view and section view) of the evolution of the Ag and Au films as a function of the laser fluence. (**a**) quartz/SnO_2_:F substrate (**b**) quartz/SnO_2_:F with 7.7 nm thickness of Au and 50 nm thickness of Ag (untreated bilayer). (**c**) Alloying process of Au/Ag films with laser fluence 0.5 J/cm^2^ and 12 ns laser pulse duration. (**d**) Dealloying process of Au/Ag films with laser fluence 0.5 J/cm^2^ and 12 ns laser pulse duration. (**e**) Nanoporous Au particles (after HNO_3_ etch of Au/Ag alloyed) with laser fluence 1.0 J/cm^2^ and 12 ns laser pulse duration.

**Figure 6:**
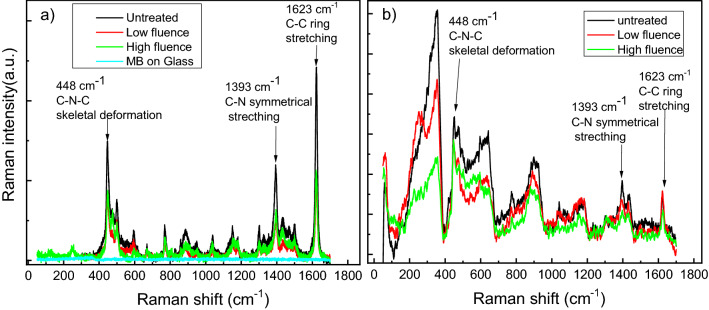
Raman spectra of methylene blue (MB) molecules on porous gold and glass substrate (**a**) and Au/Ag alloys (**b**). For (**a**) the measurements have been performed on glass (blue curve), untreated (black), high fluency region (green), Low fluency region (red). For (**b**) the measurements have been performed on untreated (black), high fluency region (green), Low fluency region (red).

Raman spectra were collected from two samples, the first is Au/Ag and the second one is the porous gold (see Fig. 5). Figure 5a–e are obtained by introducing experimental SEM images reported in the Supplementary Figs. S1a, S1b, 1b, 2b, 3b in COMSOL Multiphysics software as images. The reasons of the collection and combination of all experimental figures in Fig. 5 are: (1) to summarize the evolution of the Ag and Au films as a function of the laser fluence during the experiment as top and section views and (2) to clarify the height in nm of the nanoporous gold particles during the alloying and dealloying process using color bar. The color bar gives an indication of the height of gold nanoparticles. For example, blue color presents the small height and red colors represent the higher zone. COMSOL Multiphysics software presents a separated color bar of every figure and the identical color of Au nanoparticles and quartz/SnO_2_ does not mean a similar composition of the substrate. Each sample contains three regions that are respectively non-laser treated, treated with high power density and low power density. In order to perform SERS measurement, we used methylene blue as a Raman probe molecule, we dissolved 10^–5^ M methylene blue as a concentration in ethanol. A 10 μL volume of methylene blue was dropped into the surface and Raman measurement was carried out after 1 h drying. In order to confirm that Raman signal is coming from near-field optical enhancement generated by the metallic nanostructures and not from the optical far field, we firstly carried out a measurement on a glass substrate that showed no Raman signal, see Fig. 6.

However, the other regions for the porous gold sample, we observe high Raman intensity, this amplification is due to SERS effect generated by the porous gold nanostructures that exhibit high optical enhancement. Hot spots can be generated by the small sized-pores and near the edges of the gold nanoparticles; in turn this leads to an optical enhancement of the Raman signature, that is another factor that increases the sensitivity of SERS detection is the large specific surface area of porous nanoparticles^41^.

For the case of gold, Raman intensity of the vibrational mode of methylene blue assigned to 1623 cm^−1^ in Fig. 6a shows that untreated region exhibits two times more enhancement than the treated regions. However, in the Au/Ag sample, the same mode indicates less SERS intensity than the case of porous gold, see Fig. 6b. This decrease is observed in the three regions. The comparison between experimental and theoretical values of the enhancement factor (G), is presented in Table 1. For a comparison, the enhancement factor G for untreated porous Au and untreated Au/Ag bilayer is, also, calculated and reported in Supplementary Fig. S2 of the supporting information. As reported in the “Methods” section, we have to consider, however, that we used, for the Raman measurements, a He–Ne Laser source with a wavelength of 632.8 nm. The 1623 cm^−1^ band corresponds to 705 nm as a wavelength that is far from the two visible absorption bands located at 609 nm and 665 nm. Therefore, the Raman resonance at 705 nm was not considered in the enhancement estimation.

**Table 1:** Comparison between experimental and simulated enhancement factor (G). The method of calculation of the experimental values of the enhancement factor is detailed in the supporting information (see, also, Supplementary Fig. S3 of the supporting information).

Sample	Low fluence		High fluence	
Au porous nanostructure	Exp	Sim	Exp	Sim
	4.5 × 10^5^	5 × 10^5^	5.3 × 10^5^	8 × 10^5^
Au/Ag alloys	Exp	Sim	Exp	Sim
	6 × 10^2^	6.7 × 10^2^	1.2 × 10^2^	3.6 × 10^2^
Untreated (Au/Ag films)	Exp (with HNO_3_ etch)		Sim	
	10^4^–10^5^		2.10^4^–1.6 × 10^5^	
	Exp (without HNO_3_ etch)		Sim	
	4 × 10^2^		2 × 10^2^	

It could be important to examine plasmon resonances of the porous Au particles by direct absorption measurements, by releasing the porous Au nanoparticles on a glass slide to check their plasmon resonance spectra. Unfortunately, however, it is not possible to release them as they are attached to the FTO surface. Therefore, alternatively, first of all, we performed some indicative simulations of the absorption spectra. In fact, the plasmon resonance (SPR) spectra depends on the refractive index of the surrounding medium. In our case the porous gold were filled with air (n = 1), however, for glass medium the refractive index corresponding to 632.8 nm laser excitation is n = 1.4. We performed a simulation by changing the medium from air to glass and the results of SPR spectra for 5 nm gold nanopore radius are added and presented in Supplementary Fig. S4 of the supporting information. A clearly shift of plasmon resonance to higher wavelength is obtained as the refractive index of the medium increases. This result confirms the tuning of plasmon resonance that depends on the surrounding medium.

The COMSOL Multiphysics software is used to perform extended simulations, exploiting, in particular, the software wave optic interface covering the modeling of electromagnetic field and waves in the frequency domain. The interface proceeds, first, by formulating the Maxwell’s equation (Eq. 1):

1 $$\nabla \times \mu_{r}^{ - 1} (\nabla \times \vec{E}) - k_{0}^{2} \left( {\varepsilon - \frac{j\sigma }{{\omega \varepsilon_{0} }}} \right)\vec{E} = 0$$

being $\omega$, μ_r_, $\varepsilon$, and $\sigma$ the excitation frequency, the relative permeability (fixed to 1), the relative permittivity, and the electrical conductivity, respectively. In addition, the permittivity of free space is indicated by ε_0_ and the wave number in free space is indicated by k_0_, being k_0_ = ω/c_0_ (with c_0_ the speed of light in vacuum). In these calculations, we take the relative dielectric permittivities corresponding to the optical frequencies^42,43^ (refractive index model with *n* and *k* real and imaginary refractive indexes, respectively for the electric displacement), $\varepsilon$ = (*n* − i*k*)^2^ (in Eq. 1). Finally, in these calculations, we assume an ideal matched layer absorbing the propagating wave in the interior of the computational region and taking reflections in the interior interface. Then, the finite element method is used by the software to solve the equation and, finally, it discretizes the equation in numerically stable edge elements.

The simulated model is based on the same experimental conditions in order to well establish an accurate prediction of the electromagnetic field distribution in the porous Au nanoparticles. We carried the simulation on the pore radius effect on tuning the LSPR resonance in large electromagnetic regions (Ultraviolet, visible and near infrared regions) in order to confirm the potential of pores on the surface of gold nanospheres. There is no experimental evidence in agreement with the simulation of pores effect on tuning LSPR, however it is a complementary study for our experimental finding. The same model still gives a direct comparison with experience of the enhancement factor on Au pores nanoparticles and Au/Ag alloys. In the real system, nanoporous Au particles are formed by HNO_3_ dealloying of the alloy Au–Ag particles obtained on the FTO surface after irradiating the Ag–Au bilayer by laser fluence 1.0 J/cm^2^ that is simulated to a porous gold nanospheres with different pore radius which are, separately, pure. This explains the slightly difference of the enhancement factor in high fluence between the simulated and experimental results (Table 1). The electromagnetic effect occurs first around the porous Au nanoparticle. The local field is further enhanced, and a dipole is induced leading to the enhancement of the Raman scattering in the nanogap. Then, a mutual excitation from the system of the nanoparticles at a resonant frequency induces an enhanced apparent Raman polarizability. As a result, the simulated enhanced Raman scattered light *G* from the structure of porous Au experimentally measured for the vibrational mode located at 1623 cm^−1^ (Eq. 2) is presented as follow:

2 $$G = \left( {\frac{{I_{SERS} }}{{I_{Raman} }}} \right) \times \left( {\frac{{N_{Raman} }}{{N_{SERS} }}} \right)$$

with $I_{SERS}$ is the intensity of SERS generated by MB on Au nanoparticle, $I_{Raman}$ the intensity of Raman far field generated from glass substrate, $N_{Raman}$ the number of molecules in a laser spot generated the far-field, $N_{SERS}$ the number of molecules generated SERS signal. $N_{SERS}$ is estimated by taking in account the surface area of the porous Au nanoparticles and the number of pores and the specific area of MB molecules in porous gold nanostructures.

**Figure 7:**
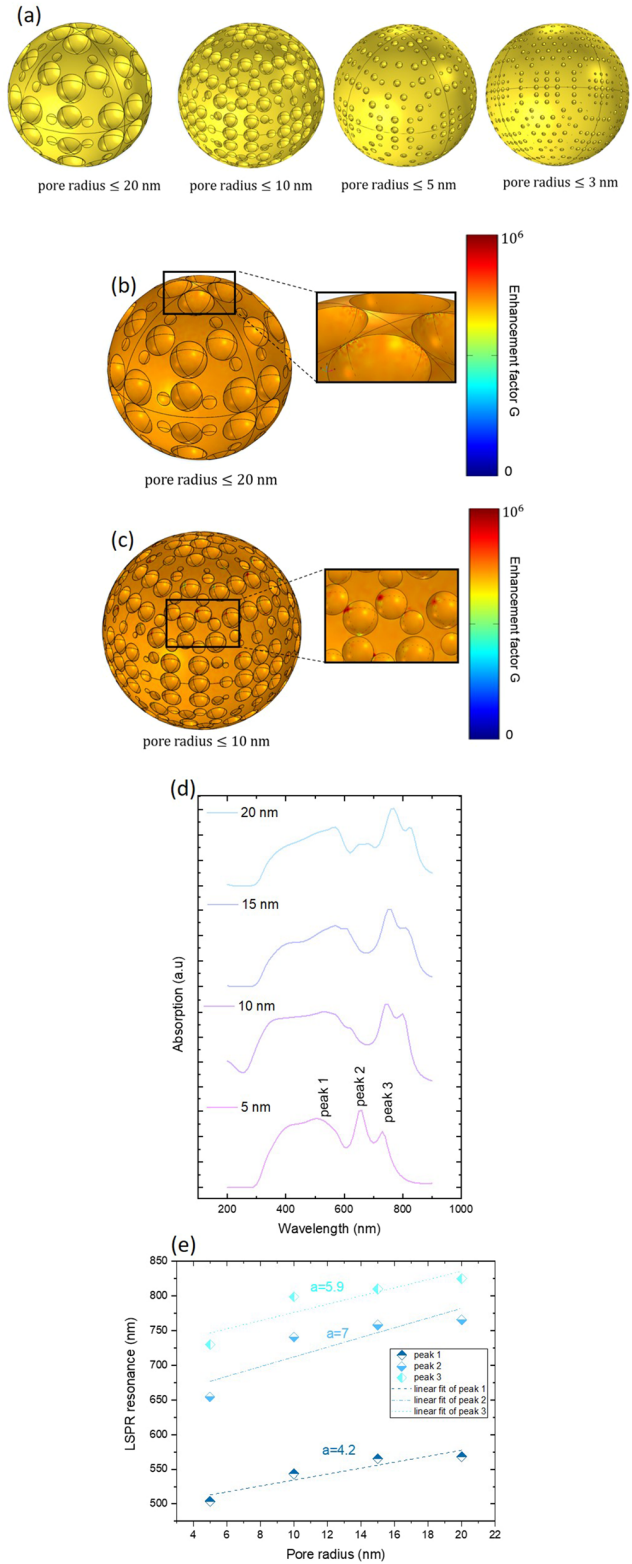
(**a**) Simulated model of porous Au with different radius. (**b**) and (**c**): simulations of electromagnetic field enhancement presenting the localization of hot spot on the porous gold nanoparticles. (**d**) Absorption of porous Au in 150 nm^2^ surface section at low laser fluence for different radii (5 nm, 10 nm, 15 nm and 20 nm). (**e**) LSPR resonance as a function of pore radius for modes in the visible and near infrared spectrum.

Nanoporous structures have been widely investigated owing to their ability to confine the electromagnetic field^44^. They exhibit extremely high enhancements thanks to their large specific surface area. Moreover, pores facilitate the transport of analytes (gas and fluid) that are important for practical analysis. When the incident beam is applied on the surface of the gold nanostructures, it can be directed deeper into the substrate through the waveguiding properties of the nanopores facilitating further Raman scattering enhancement. The transmission of incident light inside the pores of gold nanoparticles play a vital role in achieving the highest efficiency, which is critical for the ultrasensitive detection of biological and chemical species^45,46^. Therefore, we started with the investigation of pore effect as it is a potential parameter in the Au nanoparticle on the tuning of the localized surface plasmon resonance LSPR from the visible to the near infrared spectrum (200–900 nm). Figure 7a presents a schematic of the different pore radius of Au nanoparticles of 3 nm, 5 nm, 10 nm and 20 nm. The modeled pores are randomly distributed on the surface of the gold nanospheres with different gap. Figure 7b,c report, as example, simulations of electromagnetic field enhancement presenting the localization of hot spot on the porous gold nanoparticles with different size.

**Figure 8:**
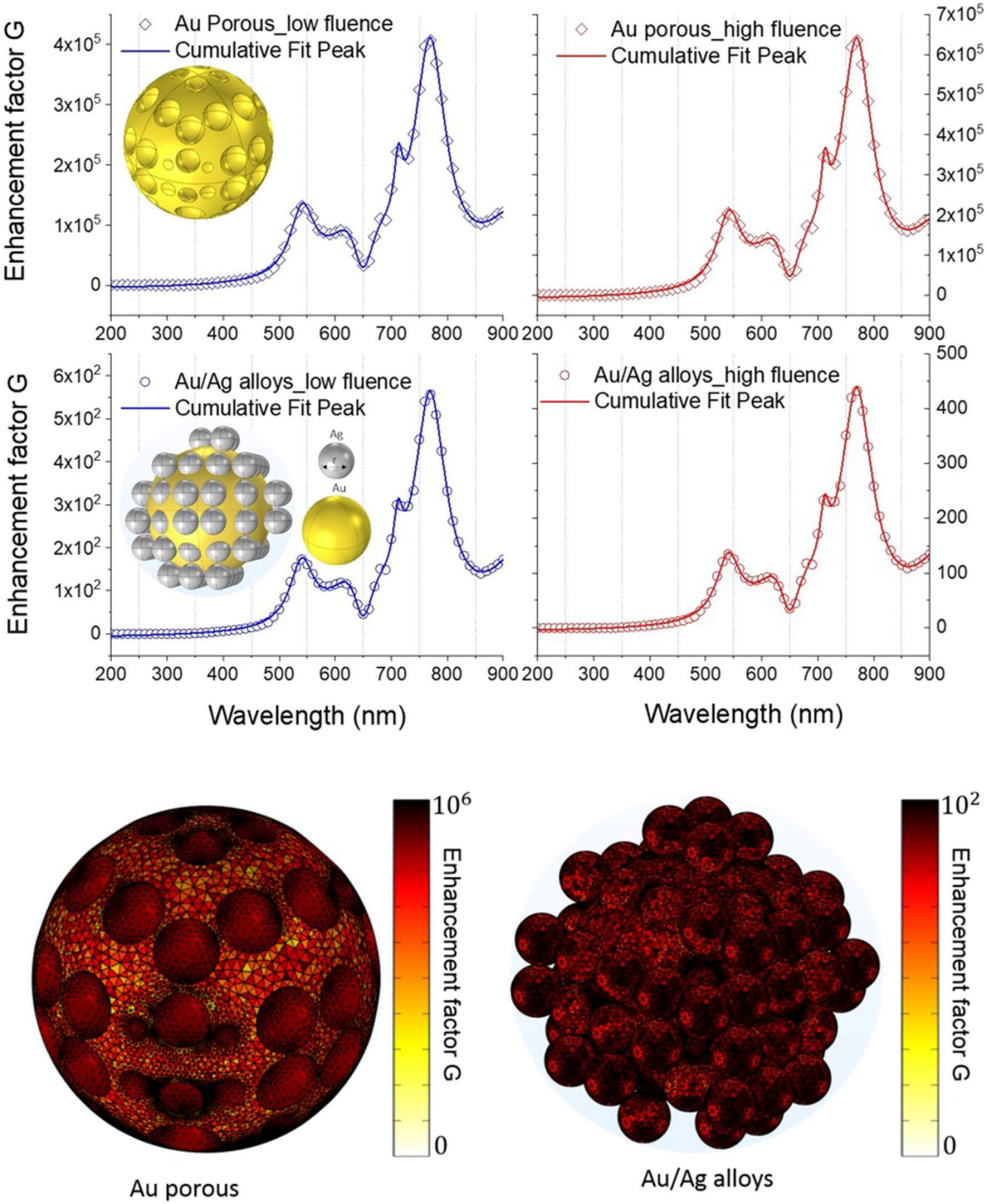
Up: enhancement factor G in Au/Ag alloys and porous Au for low and high laser fluence. Down: simulations of electromagnetic field enhancement presenting the localization of hot spot on the porous gold nanoparticles and in Au/Ag alloys.

The absorption of porous gold is depicted in Fig. 7d. The spectrum presents different modes in the Ultraviolet (UV), visible (V) and near infrared (NIR) regions. As the pore radius of Au nanospheres increases, the splitting mode for 3 nm in the visible range starts shouldering for 10 nm, 15 nm and 20 nm. However, a broadening of the peak 1 appears as the radius decreases. Example for pore radii $\le 20 \;{\text{nm}}$, that will be considered in detail in the next enhancement factor simulation, the modes are located at 563 nm, 644 nm, 685 nm, 771, nm and 828 nm. More detail about the LSPR resonance of three selected peaks is studied in Fig. 7e. A very clear redshift is detected for all modes as the radius increases with different slope denoted *a* (peak 1 with *a* = 4.2, peak 2 with *a* = 7, peak 3 with *a* = 5.9). Minimal slope of LSPR is obtained between 500 and 550 nm with 4.2 and the maximum shift of LSPR are obtained between 650 and 775 nm. Between 725 and 800 nm, LSPR shows similar behavior with a remarkable redshift of resonance. The LSPR resonance tuning is expected as the pore radius changes since they are very sensitive to the dimension and optical properties of the surrounding medium. The study of pore radius effect on the LSPR resonance is potential for tuning the sensitivity of gold porous nanoparticles. In order to compare between the different obtained regions and sample structures, we carried out an estimation of the optical enhancement by taking the Raman intensity at 1623 cm^−1^, see the Table 1. We simulate two configurations: (1) the Au porous nanostructure. (2) the Au/Ag alloys nanostructure (see the plots of the enhancement factor in Fig. 8) for low and high laser fluence. The enhancement factor is estimated on the surface of a single gold nanosphere but considering without (configuration 1) and with (configuration 2) silver nanoparticles. As assumptions, we considered that (a) the silver nanoparticles are randomly distributed on the surface of gold nanosphere with pore radii $\le 20 \,{\text{nm}}$ and (b) the model of a unique gold nanosphere and (c) a two different laser pulse intensity per unit area for low and high fluence. The resulting enhancement spectrum of Au/Ag alloys and Au porous nanostructure presents 5 modes (two in the visible spectrum and three in the NIR spectrum).

The LSPR modes are located at (533 nm, 647 nm, 712 nm, 769 nm, 921 nm). A direct comparison between these LSPR modes obtained in Fig. 8 and LSPR modes obtained in Fig. 7d for pore radii $\le 20 \,{\text{nm }}$ proves the same localization of these modes with a small shift. Besides, our result confirms that Au porous nanostructure has the highest enhancement factor compared to Au/Ag alloys structure for low and high fluence. A good agreement between the enhancement factor on the surface of the gold nanosphere in the simulated and experimental analysis is noticed (see, also, Supplementary Fig. S1 in the supporting information).

In this regard, Fig. 8 reports, also, simulations of electromagnetic field enhancement presenting the localization of hot spot on the porous gold nanoparticles and in Au/Ag alloys. Additional simulations (concerning smaller porous Au particles) are reported in Supplementary Fig. S5 of the supporting information Our finding confirms that nanoporous Au acts simply as a coupler which converts efficiently the incoming radiation into surface waves and therefore intense field enhancement is obtained.

To conclude, we observe that the experimental Raman spectra obtained in this study are the far-field in different point on the substrate. We did not use TERS spectroscopy (that gives the topography) for mapping the substrate. However, we added examples of simulations of electromagnetic field enhancement presenting the localization of hot spot on the porous gold nanoparticles, see Figs. 7b,c, 8, and Supplementary Fig. S5 of the supporting information. Moreover, gold nanoparticles and porous gold nanoparticles with same size exhibit field enhancement but with quantitatively different electromagnetic field enhancement. The nanoporous gold particles possess a much higher surface-to-volume ratio than bulk gold nanoparticles. As the number of nanoscale pores increased and the gap between them decreased, the intensity of the multimodes of surface plasmon gradually decreased due to the presence of sharp tips at the nanopore surfaces (Fig. 7a, gold pore radii lower than 20 nm). Therefore the big part of the field enhancement comes from the pores of gold nanoparticles.

## Conclusions

As a conclusion, we have succussfully fabricated Au/Ag alloys and porous Au nanoparticles during irradiation of Ag–Au bilayer deposited on FTO (SnO_2_:F) substrate by laser fluence equal to 0.5 J/cm^2^ or 1.0 J/cm^2^ with 12 ns laser pulse duration. We have studied the corresponding samples through both experiments and simulations. We investigated SERS enhancement tunability in Ag/Au alloys and porous Au nanostructures and LSPR tunability in porous Au nanostructures. We have analyzed the LSPR dependance on the radius of Au and Ag nanostructre. We also analyzed the dependence of the enhancement factor G on the laser fluence and on the obtained nanoisland (Au/Ag alloys and porous Au). Towards that end, we have developed using a finite element method a model that confirms the experimental enhancement factor obtained. Finally, we confirmed that porous Au nanoparticles are very promissing for SERS applications as they confine efficiently the incoming radiation.

## Methods

The set of samples were prepared starting from quartz/FTO (soda-lime/SnO_2_:F) pieces as substrate (~ 85% transmittance for the quartz/FTO system in the 500–1000 nm wavelength range, ~ 8.6 Ω/sq resistivity for the FTO layer^8^). The quartz/FTO substrate was chosen since: (i) it presents a high transmittance at the laser wavelength of 532 nm which is used for the following alloying and dewetting processes of the deposited Au/Ag bilayers (avoiding, so, FTO damage during the laser irradiations, whereas, for example, furnace annealing processes above 400 °C structurally damage the FTO layer determing dramatic increase in its resistance, see Ref.^8^ for details); (ii) it is a standard substrate used as a commercial transparent and conductive oxide already exploited in specific industrial applications so that it is a suitable supporting substrate for the nanoporous Au particles in view of specific functional applications.

The fabrication procedure for the alloy AuAg particles and for the Au nanoporous particles is detailed reported in Ref.^47^. Here we report the basic steps according to Ref.^47^: Au and Ag depositions were carried out on the quartz/FTO substrate slides using a Emitech K550X Sputter coater apparatus, clamping the substrates against to the cathode located straight in front of the source (99.999% purity target). The electrodes were laid at a distance of 40 mm under Ar flow, keeping a pressure of 0.02 mbar in the chamber. The Au and Ag depositions were performed sequentially. First the Au layer was deposited with thickness x_Au_ = (7.7 ± 0.5 nm), then the Ag layer with thickness x_Ag_ = (50.0 ± 3.0) nm on the previously deposited Au layer. Alloying and dewetting processes of the Au/Ag bilayers on the FTO substrate were performed by laser irradiations (one pulse) using a pulsed (12 ns) Nd:yttrium aluminum garnet YAG laser, operating at 532 nm (Quanta-ray PRO-Series pulsed Nd:YAG laser). The spot laser has a circular shape of 4 mm in diameter. The laser intensity profile is Gaussian, and it is characterized by a full width at half maximum of 1 mm. 97% of the highest laser intensity is maintained within a circular area of 600 μm in diameter centered at the maximum of laser intensity. Two samples were prepared by using two different laser fluence: 0.5 J/cm^2^ and 1.0 J/cm^2^ (the error in the fluence measurement is 25 mJ/cm^2^). The laser-induced alloying and dewetting processes of the Au/Ag bilayer leads to the formation of Au_13_Ag_87_ (at%) alloy particles supported on the FTO substrate. The Au and Ag atomic % ratio was checked by Rutherford–Backscattering Spectrometry analyses were by using 2 meV ^4^He^+^ backscattered ions with a scattering angle of 165°.

Then, the dealloying process of the alloy particles, to selectively remove the Ag atoms, was performed by 70%-HNO_3_ solution. In particular, a 50 μl drop of the solution was placed on the sample surface by a micropipette and, after 20 min, it was removed by the micropipette. Finally, the sample was rinsed in de-ionized water for 30 min so to completely eliminate HNO_3_ residuals within the nanopore channels.

Scanning Electron Microscopy (SEM) analyses were performed by a Zeiss FEG-SEM Supra 25 Microscope operating at 5 kV. The SEM images were analyzed by the Gatan Digital Micrograph software. In particular: (i) to extract from the SEM images the average diameter < D > of the particles, several SEM images per sample were considered, each containing several particles. Then, for each image we set a threshold on the brightness of the image, so that the bright regions in the images, with intensity value 1, represent the metal particles and the dark regions, with intensity value 0, represent the supporting substrate. The diameter D of a particle is evaluated as the diameter of the smaller circle inscribing the particle. The mean value < D > of the particles for each sample has been extracted by averaging on several particles per sample, with the error the standard deviation on the mean value. (ii) To extract from the SEM images the fraction of the surface porosity (F%) of the nanoporous Au particles, high magnification SEM images were acquired over the surface of several particles per sample. For each image we set a threshold on the brightness of the image, so that the bright regions in the images, with intensity value 1, represent Au (the ligaments) and the dark regions, with intensity value 0, represent the pores. So, F% was obtained by dividing the total area of the pores in the image by the total area of the image (and expressed as percent). A mena value was evaluatred by averaging on several particles and associating an error arising from the statistical averaging procedure.

Raman spectra were collected using LabRam HR evolution spectrometer (LabRAM HR800, Horiba Scientific, Villeneuve-D’Ascq, France) in a backscattering geometry with a spectral resolution of 0.3 cm^−1^ at ambient temperature. A He–Ne laser of λ = 632.8 nm and a power level of 0.4 mW were used. The lower power density has been chosen in order to prevent any thermal effect. An objective of 50× with a numerical aperture of 0.5 was used.

## Supplementary Information

Supplementary Information.
