# Supplementary material for: High optical enhancement in Au/Ag alloys and porous Au using Surface-Enhanced Raman spectroscopy technique
Source: Sci Rep. 2021 Feb 25;11:4714. doi: 10.1038/s41598-021-84093-0 (PMC7907086; doi:10.1038/s41598-021-84093-0)
Supplement: Supplementary file 1 — Supplementary Information. [file 41598_2021_84093_MOESM1_ESM.docx]

**Supporting information**

**High optical enhancement in Au/Ag alloys and porous Au using Surface-Enhanced Raman spectroscopy technique**

C. Awada*^1^, C. Dab^2^, M. G. Grimaldi^3^, A. Alshoaibi^1^, F. Ruffino*^3^

AUTHOR ADDRESS

^1^Department of Physics, College of Science, King Faisal University, P.O Box: 400, Al-Ahsa 31982, Saudi Arabia.

^2^Département de chimie, Université de Montréal, Montréal QC H3C 3J7, Canada.

^3^Dipartimento di Fisica e Astronomia "Ettore Majorana"-Università di Catania and MATIS CNR-IMM, via S. Sofia 64, 95123 Catania-Italy

*[cawada@kfu.edu.sa](mailto:cawada@kfu.edu.sa)

*[francesco.ruffino@ct.infn.it](mailto:francesco.ruffino@ct.infn.it)


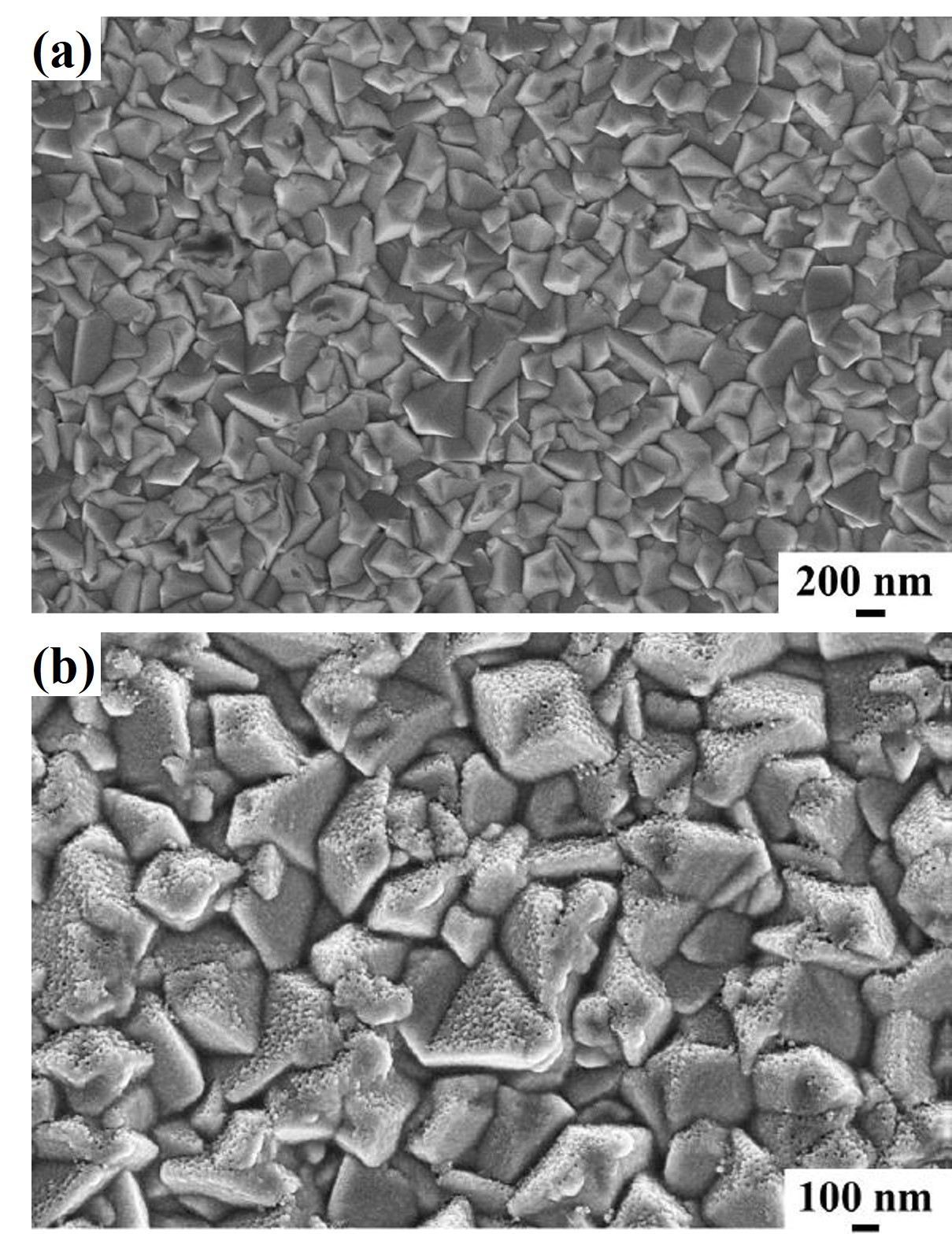


Figure S1. (a) Representative SEM image of the starting surface of the FTO layer (on quartz) used as supporting substrate for the nanoporous Au particles. (b) Plan-view SEM image of the FTO surface after being covered by the Au and Ag films.

Figure S2. Enhancement factor G for (a) untreated porous Au and (b) untreated Au/Ag bilayer.

**Calculation of enhancement factor**

$$G=\frac{I_{SERS}}{I_{Raman}}\frac{N_{Raman}}{N_{SERS}}$$

with $I_{SERS}$ is the intensity of SERS generated by MB on Au nanoparticle, $I_{Raman}$ the intensity of Raman far field generated from glass substrate. $N_{Raman}$ number of molecules excited by a laser spot with a diameter of 1.5 μm. The number of molecules in a drop of MB (10^-5^ M) with a volume of 10 μL is around 6 x 10^13^ molecules. The drop can be dispersed into a disc with a diameter of 10 mm. The ratio of the laser spot diameter to the dispersed surface gives $N_{Raman}$~10^5^ molecules.

$N_{SERS}$ is the number of molecules excited by the hotspot in the pores. In order to estimate them, we performed a statistical study of the SEM images that contain the porous sphere by using Watershed mark in order to estimate the number of pores by a surface unit, see Fig. S3. The number of pores is estimated to be around 400 pores with the average surface of 5 nm^2^. If each pore contains one molecule, N_SERS_ could contain around 4x10^2^ molecules. It is important to mention that not all the pores can generate hotspots. Therefore, the number of molecules susceptible to generate SERS signal is estimated with an order of magnitude of 10^2^, consequently, $\frac{N_{Raman}}{N_{SERS}}={10}^{3}$. For the porous gold, from Fig. 6, we can determine $\frac{I_{SERS}}{I_{Raman}}=500$. Finally, we can conclude an enhancement factor G~10^5^-10^6^. For the non-porous nanostructures, the estimation is more simple, the ratio $\frac{N_{Raman}}{N_{SERS}}$ depends on the ratio of the laser spot and the surface of nanoparticles.

We can consider, ultimately, that gold nanoparticles and porous gold nanoparticles with same size exhibit field enhancement but with quantitatively different electromagnetic field enhancement. The nanoporous gold particles possess a much higher surface-to-volume ratio than bulk gold nanoparticles. As the number of nanoscale pores increased and the gap between them decreased, the intensity of the multimodes of surface plasmon gradually decreased due to the presence of sharp tips at the nanopore surfaces (see Fig. 7(a), gold pore radii lower than 20 nm). Therefore the big part of the field enhancement comes from the pores of gold nanoparticles.


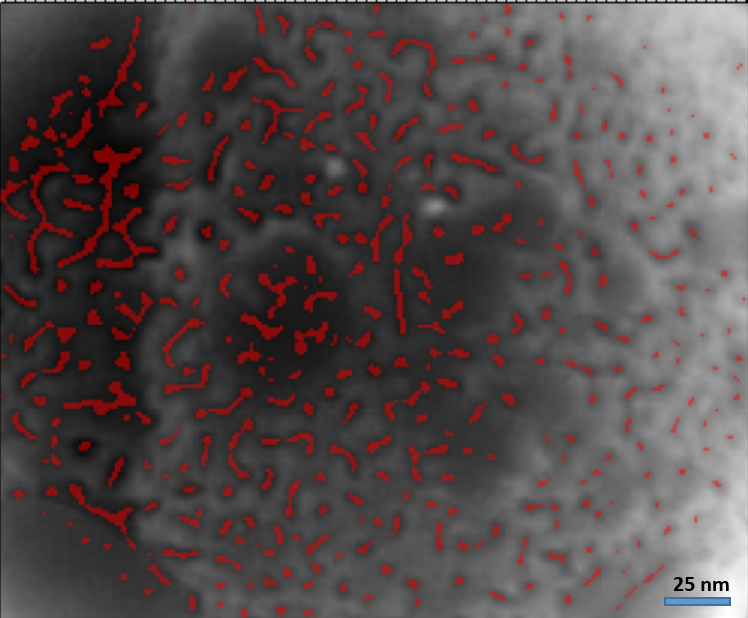


Figure S3. A SEM image of a porous sphere masked by watershed in order to estimate the number of pores by surface unit.

Figure S4. Simulated absorption spectra of nonoporous gold (5 nm pore radius) in air and in glass presenting the tuning of surface plasmon resonance depending on the surrounding medium.


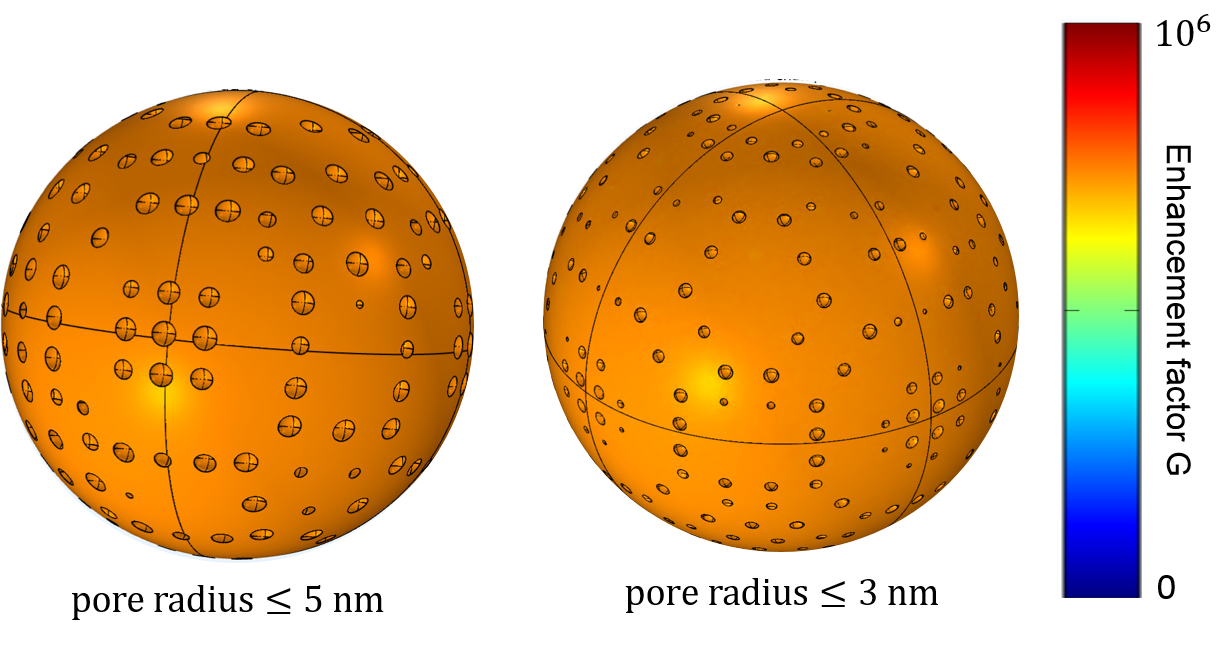


Figure S5: Simulated enhancement factor for porous Au particles with different radius.
